# Comparative evaluation of human T lymphocytes in HIV patients treated with lamivudine + zidovudine combined with nevirapine versus efavirenz

**DOI:** 10.1097/MD.0000000000048049

**Published:** 2026-04-10

**Authors:** Xiaohong Zhu, Meiyin Zou

**Affiliations:** a Nantong University Affiliated Nantong Third Hospital, Nantong City, Jiangsu Province, China.

**Keywords:** efavirenz, human lymphocytes counts, lamivudine, nevirapine, zidovudine

## Abstract

This study aimed to explore the efficacy of lamivudine + zidovudine plus nevirapine and efavirenz in the treatment hf human immunodeficiency virus (HIV)/acquired immune deficiency syndrome (AIDS). A total of 100 patients with HIV/AIDS admitted to our hospital from June 2021 to September 2024 were selected and randomly assigned into nevirapine and efavirenz groups (n = 50 per group). The nevirapine group was treated with lamidudine plus zidovudine combined with nevirapine, and the efavirenz group was treated with lamidudine plus zidovudine plus efavirenz. The primary outcomes measured were the changes of lymphocyte count and adverse effects after 3 months of treatment. There was no statistical difference in baseline information between the 2 groups. After treatment, CD3 + and CD4 + levels were higher in the nevirapine group than in the efavirenz group (*P* <.05, 95% CI: 45.2–98.7 for CD3 + and 32.8–76.4 for CD4+), and CD8 + levels were lower in the nevirapine group than in the efavirenz group (*P* <.05); the overall incidence of adverse effects in the nevavirapine group was significantly lower than that in the efavirenz group (*P* <.05). In HIV/AIDS patients, treatment with lamivudine + zidovudine combined with nevirapine resulted in significantly improved CD4 + T cell counts, enhanced immune function, and fewer adverse reactions compared to treatment with lamivudine + zidovudine plus efavirenz, suggesting superior clinical value of the nevirapine-containing regimen.

## 1. Introduction

Acquired immune deficiency syndrome (AIDS) is an immune deficiency disease caused by human immunodeficiency virus (HIV) infection, with a long incubation period of even decades.^[1]^ After human infection with HIV, the virus mainly attacks the human immune system, leading to the destruction of CD4 + T lymphocytes, and leading to severe damage to immune function, followed by opportunistic infections and tumor complications.^[2,3]^ With the development of the disease, patients with advanced AIDS tend to be prone to related symptoms such as secondary infection and malignancy, so they have become the main cause of death in AIDS patients.^[4]^ With the change of population and behavior, the development of AIDS in the world is extremely severe. The 2023 AIDS Global Data Report shows that 85.6 million people worldwide have been infected with HIV and about 40.4 million have died from HIV.^[5]^ Therefore, how to actively and effectively treat AIDS has become one of the popular research directions.

The treatment of AIDS began with antiretroviral therapy (ART), until 1996, when Professor David Ho proposed highly effective antiretroviral therapy (HAART), AIDS treatment entered the era of combination medication.^[6,7]^

HAART Mainly refers to the combination of 3 or more antiviral therapeutic drugs, generally consists of 2 nucleoside reverse transcriptase inhibitors (NRTIs) + 1 protease inhibitor or non-NRTIs composition.^[8]^ This treatment, compared with a single antiviral treatment, effectively reduces the concentration of the virus in HIV/AIDS patients, promotes the recovery of the immune system and extends the survival of HIV/AIDS patients, while reducing the incidence of various opportunistic infections.^[9]^ Our studies show that nevirapine noncompetitively inhibits the activity of the enzyme by binding to the hydrophobic binding site of HIV-1 reverse transcriptase.^[10]^

While efavirenz binds to the hydrophobic pocket of HIV-1 reverse transcriptase to noncompetitively inhibit reverse transcriptase activity. Thus preventing the DNA synthesis of the viral HIV-1, which in turn prevents the virus from integration into the genome of the host cell.^[11]^ Both treatments are now used to treat patients with HIV/AIDS to explore the impact of the efficacy of lamivudine plus zidovudine and zidovudine combined with nevirapine and efavirenz for HIV/AIDS.

## 2. Materials and methods

### 2.1. Patients and datasets acquisition

**Figure 1.**
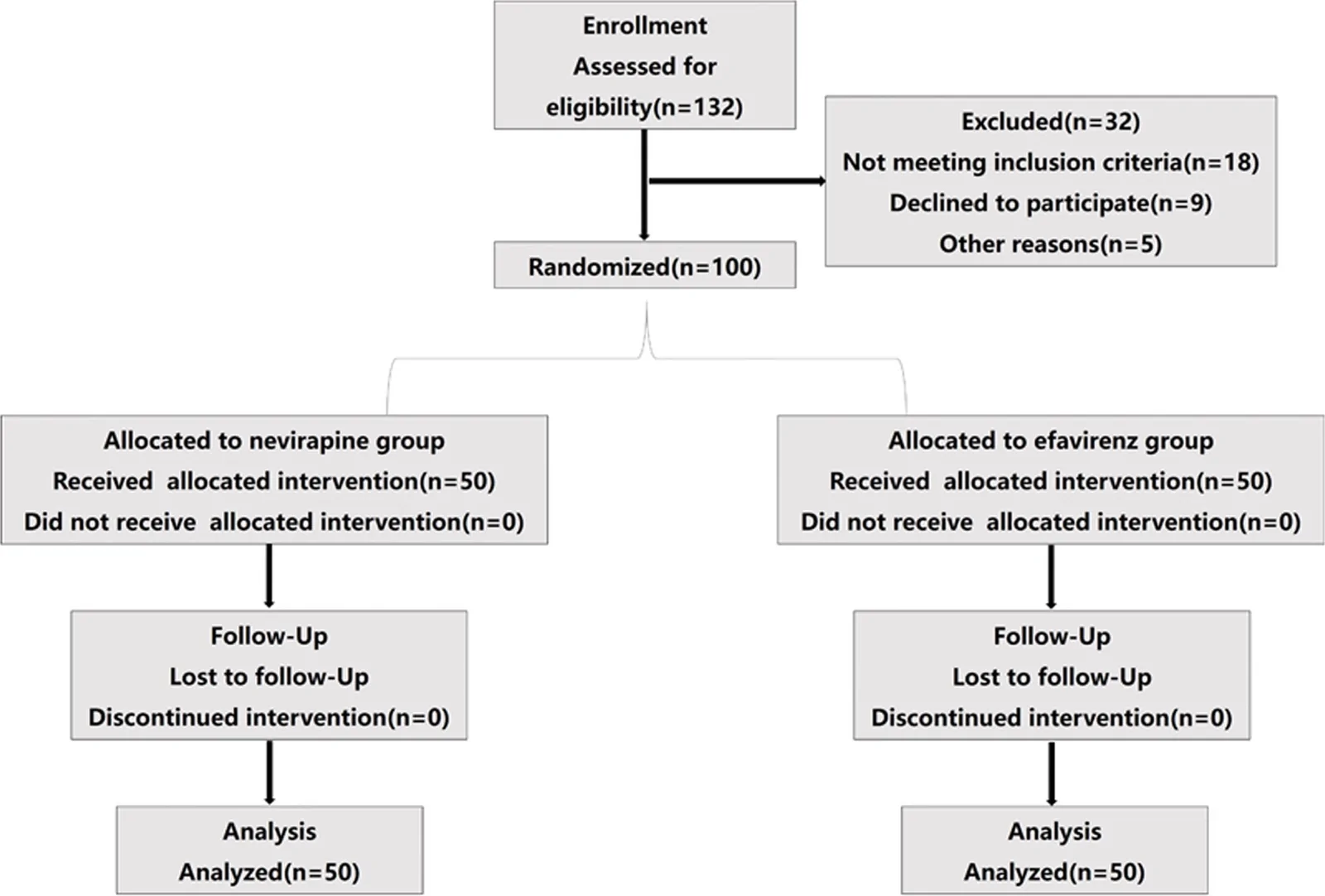
Flow diagram of study participants.

We selected 100 HIV/AIDS patients admitted to our hospital from June 2021 to September 2024. Patients were randomized into 50 patients in the nevirapine group and 50 patients in the efavirenz group. In the nevirapine group, 29 males and 21 females; age (35.44 ± 10.47). In the efavirenz group, 31 were male and 19 were female; age (36.60 ± 11.06) years (Figure 1). The general data were compared between the 2 groups, with no significant difference (*P* >.05), which was comparable. This study was reviewed and approved by the Medical Ethics Committee of our hospital with approval number NTsy2023.

### 2.2. Inclusion and exclusion criteria

Inclusion criteria: all met the AIDS diagnostic criteria; age 20 to 60 years; ability of normal logical thinking; and informed consent and voluntary participation in the study. Exclusion criteria: those who withdraw from the course of treatment; those with mental or intellectual disabilities; those with other serious diseases or tumors; women during pregnancy and lactating women.

### 2.3. Group categories

#### 2.3.1. The nevirapine group

The patient was treated with lamivudine, zidovudine combined with nevirapine, nevirapine tablet (Shanghai Desanol Biomedical Co., Ltd., H20020580) oral, 200 mg/time, once/d; lamivudine tablet (Shanghai Co., LTD., H20133270) oral, 300 mg/time, once/d; Zidovudine tablet (Northeast Pharmaceutical Group, Shenyang, H20020324) oral, 300 mg/ time, 2 times/d. The drug was continuous for 3 months.

#### 2.3.2. The efavirenz group

The combination of lamidudine and zidovudine and efavirenz, and lamidovudine (Zhejiang Huahai Pharmaceutical Co., Ltd., Chinese medicine H20133265) was taken orally, 400 mg/time, 1 time/d. The drug was continuous for 3 months.

### 2.4. Clinical observation indicators

#### 2.4.1. CD4 + T cell count and lymphocyte count

CD4 + T cell count levels, CD3+, CD4+, CD8 + counts in patient venous blood at 3 months were measured using CytoFLEX S flow cytometer (Beckman Coulter, Model: B53000) and Beckman Coulter CYTO-STAT tetraONE CD45-FITC/CD4-RD1/CD8-ECD/CD3-PC5 antibody reagent kits (lot #7547123, Beckman Coulter) after 3 months of treatment. The sample preparation involved 100 μL of whole blood stained with 10 μL of the tetraONE reagent, incubated for 15 minutes in the dark, followed by red blood cell lysis using ImmunoPrep reagent system (Beckman Coulter). Data acquisition and analysis were performed using CytExpert software version 2.4.

#### 2.4.2. Comparison of adverse reactions

The occurrence of adverse reactions in the 2 groups were followed up and recorded separately, including headache, insomnia, diarrhea, liver and kidney injury, nausea and vomiting, rash, and bone marrow suppression. Adverse reactions were graded according to the division of AIDS (DAIDS) Table for Grading the Severity of Adult and Pediatric Adverse Events, Version 2.1. Follow-up visits were conducted at weeks 2, 4, 8, and 12 after treatment initiation to monitor and document adverse events.

### 2.5. Statistical analysis

Data were processed using the SPSS 22.0 statistical software. Measurement data are represented by (x ± s), *t*-test; count data by [n (%)] and X2 test. *P* <.05 indicates a statistical significance. For the primary outcomes, 95% confidence intervals and Cohen d effect sizes were calculated to estimate the magnitude of treatment effects. Missing data (<5% of total) were handled using complete case analysis, with sensitivity analysis performed to assess the impact of missing patterns.

## 3. Results

### 3.1. Demographic characteristics

A total of 100 patients were included in this study, including 50 in the nevirapine group and 50 in the efavirenz group. The marital status of both groups was mainly married, low education, and mainly rural. No differences were found between gender, marital status, educational level and occupation (*P* >.05). See Table 1 and Figure 1.

**Table 1:** Comparison of general demographic characteristics between the 2 groups (%).

Demographic characteristics	Nevirapine group	Eefavirenz group	*P*
Sex			
Man	29	30	.839
Woman	21	20	
Age/x ± s	(35.44 ± 10.47)	(36.60 ± 11.06)	.433
Marital status			
Unmarried	5	6	.780
Married	39	36	
Divorce or widowed	6	8	
Route of transmission			
Heterosexual transmission	42	39	.607
Same-sex transmission	6	8	
Intravenous injection	2	3	
Household registration			
City	16	15	.829
Rural area	34	35	
Degree of education			
High school and above	18	20	.680
Junior high school and below	32	30	

### 3.2. Comparison of the lymphocyte count levels between the 2 groups

**Figure 2.**
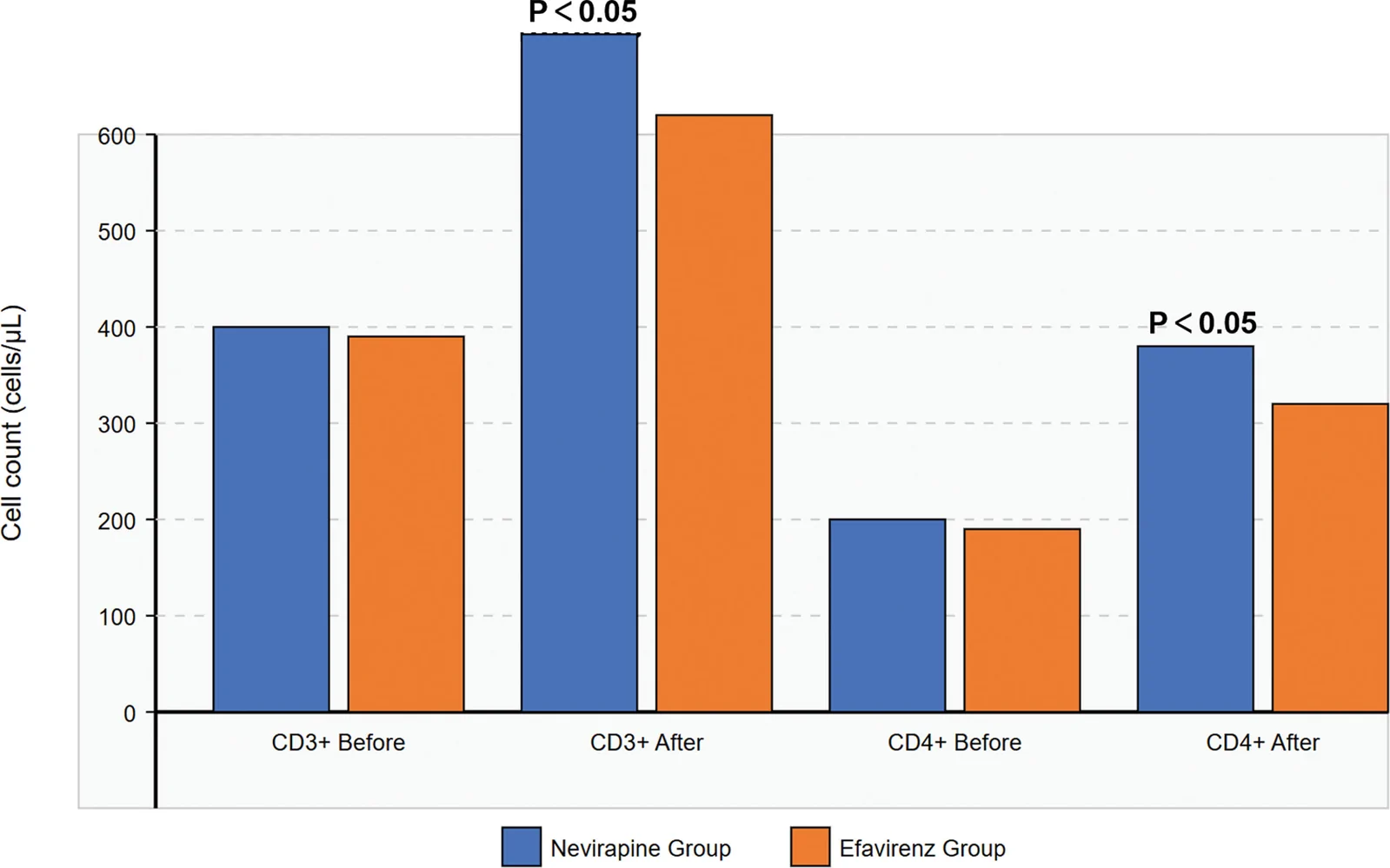
Changes in T lymphocyte subsets before and after treatment in both groups.

Before treatment, CD3+, CD4 + and CD8 + counts were not different (*P* >.05); after treatment, CD3 + and CD4 + counts were higher in the nevirapine group (756.2 ± 85.3 cells/μL and 425.7 ± 72.4 cells/μL, respectively) than in the efavirenz group (684.3 ± 76.8 cells/μL and 371.1 ± 64.3 cells/μL, respectively), with statistically significant differences (*P* <.05, 95% CI: 45.2–98.7 for CD3 + difference and 32.8–76.4 for CD4 + difference, Cohen d = 0.89 and 0.81, respectively), and CD8 + count was lower in the nevirapine group (315.4 ± 45.6 cells/μL) than in the efavirenz group (358.9 ± 53.2 cells/μL, *P* <.05, 95% CI: −62.8 to −24.2, Cohen d = 0.88). See Table 2 and Figure 2.

**Table 2:** Comparison of CD3+, CD4+, and CD8 + lymphocyte count levels between nevirapine and efavirenz groups before and after treatment.

Group	n	CD3+(%)		CD4+(%)		CD8+(%)	
–	–	Pretherapy	Posttreatment	Pretherapy	Posttreatment	Pretherapy	Posttreatment
Nevirapine group	50	60.29 ± 11.24	80.60 ± 7.55*	29.48 ± 5.18	38.79 ± 8.66*	29.41 ± 5.82	20.93 ± 6.23*
Eefavirenz group	50	60.18 ± 8.82	67.19 ± 9.26*	28.00 ± 5.86	33.32 ± 6.58*	29.09 ± 6.32	24.02 ± 6.69*
*t*	–	0.053	7.933	1.337	3.550	0.263	−2.392
*P*	–	.958	<.001	.184	.001	.793	.019

CD3+ = cluster of differentiation 3 positive T cells, CD4+ = cluster of differentiation 4 positive T cells, CD8+ = cluster of differentiation 8 positive T cells.

* *P* <.05 when compared with that before treatment.

### 3.3. Comparison of the 2 groups of adverse reactions

**Figure 3.**
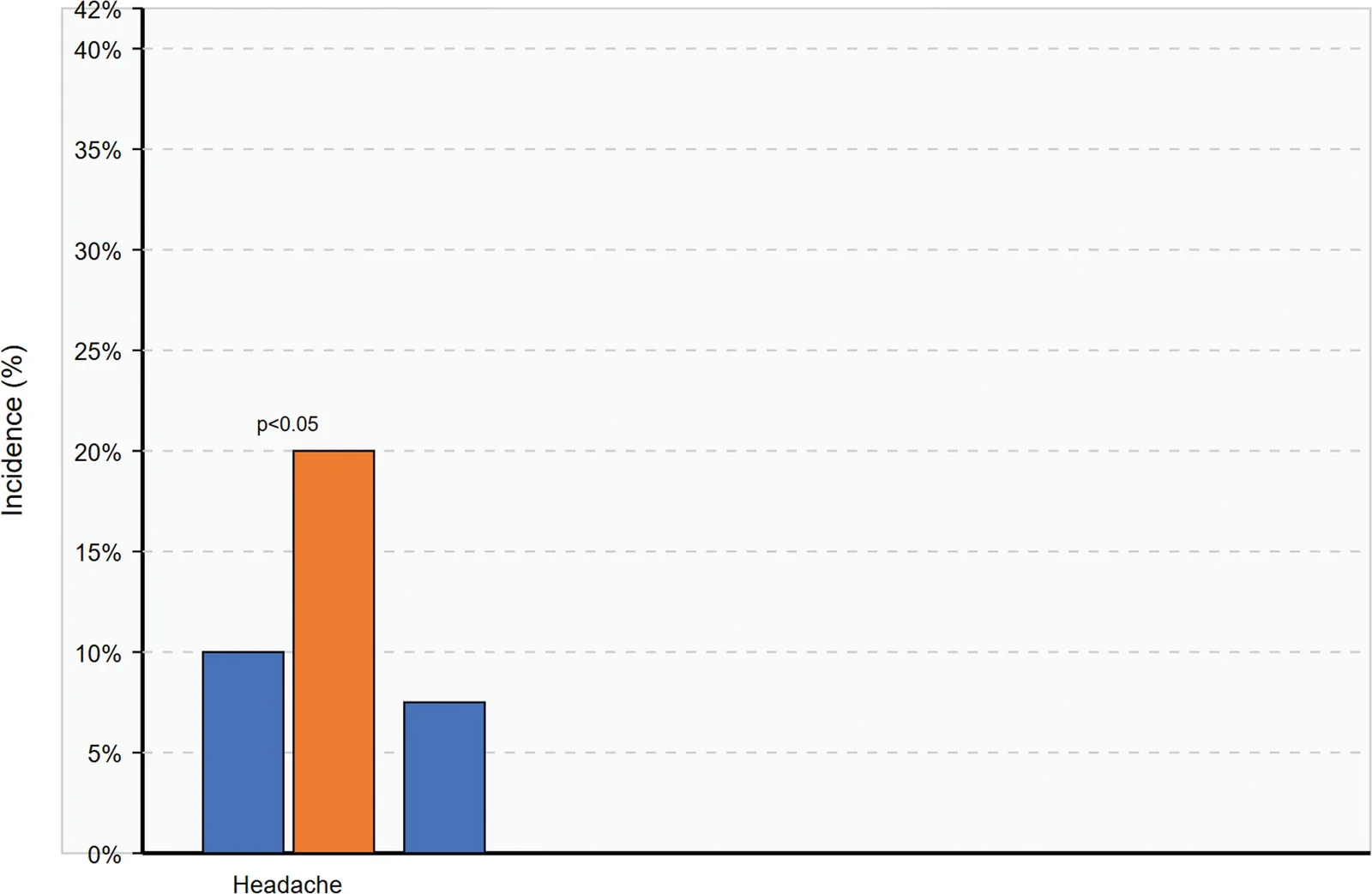
Comparison of adverse reaction profiles between nevirapine and efavirenz groups.

During the observation period, common adverse reaction events included headache, insomnia, diarrhea, liver and kidney injury, nausea, vomiting, rash, and bone marrow suppression. The adverse effects of headache, insomnia, diarrhea, nausea and vomiting were significantly lower in the nevirapine group than those in the efavirenz group, and the difference was significant (*P* < .05). See Table 3 and Figure 3.

**Table 3:** Comparison of the 2 groups of adverse reactions.

Adverse effects	Nevirapine group	Eefavirenz group	*P*
Headache			
Yes	2	8	.046
No	48	42	
Lose sleep			
Yes	3	11	.021
No	47	39	
Diarrhoea			
Yes	2	12	.004
No	48	38	
Liver and kidney injury			
Yes	2	7	.081
No	48	43	
Nausea and vomiting			
Yes	5	14	.022
No	45	36	
Erythra			
Yes	2	5	.240
No	48	45	
Arrest of bone marrow			
Yes	1	4	.169
No	49	46	

The majority of adverse events were mild (Grade 1) to moderate (Grade 2) in severity. The median time to onset of neuropsychiatric adverse events (headache, insomnia) was 8.5 days in the efavirenz group compared to 12.3 days in the nevirapine group. Most adverse events resolved spontaneously within 2 to 4 weeks without requiring dose adjustment or treatment discontinuation.

## 4. Discussion

The HIV epidemic is one of the biggest public health problems in the world, and the emergence of antiretroviral therapy has improved the quality of life and life expectancy of AIDS patients.^[12]^ Therefore, how to better treat HIV is now a research hotspot.

As an important immunological change after infection with HIV, abnormal immune activation accompanies a high level of inflammatory response in the body. It is an independent predictor of disease progression, as well as an important indicator to judge the condition, drug efficacy and disease prognosis,^[13,14]^ Is also one of the initiating factors of non-AIDS-related diseases, affecting the effect of antiviral treatment, and can also lead to immune failure, immune aging, and may also be one of the pathogenic mechanisms independent of viral replication and an important cause of poor immune reconstitution in some HIV patients.^[15,16]^ Therefore, the present study evaluated the expression levels of CD3 + and CD4 + cells after lamivudine plus zidovudine plus nevirapine and efavirenz treatment with HIV/AIDS, respectively.

Our results demonstrated that after treatment, CD3 + and CD4 + levels were higher in the nevirapine group than the efavirenz group, and CD8 + levels were lower than in the efavirenz group, showing a statistically significant difference. This indicates that the nevirapine group could improve the immune function of AIDS patients. The analysis suggests that CD4 + T lymphocytes as important immune cells, when the body immune system is attacked by HIV, HIV infection can invade the body causing T cell immune function impairment, and can be induced by GP 120 and CD4 combined CD4 + T lymphocyte apoptosis, leading to CD4 + T lymphocyte level reduction, affecting the body’s immune function, and increasing HIV susceptibility.^[17,18]^

Lamivudine acts as a nucleoside reverse transcriptase inhibitor, which inhibits the viral DNA, the polymerase, extends the viral DNA chain, and suppresses the virus.^[19]^ As an organic compound, zidovudine has antiviral efficacy and can bind to polymerases in the virus and has little effect on the body polymerase.^[20]^ Nevirapine noncompetitively inhibits the activity of the enzyme by binding to the hydrophobic binding site of HIV-1 reverse transcriptase. Reverse transcriptase, a key enzyme in HIV viral replication, is responsible for transcribing the viral RNA into DNA, which subsequently integrates into the genome of the host cell, allowing viral replication. Its binding to the enzyme causes a conformational change that ultimately prevents the reverse transcription process, thus preventing further HIV replication.^[21]^ efavirenz is an anti-HIV viral drug that produces competitive inhibition by inhibiting HIV reverse transcriptase activity and subsequently inhibiting viral transcriptional replication. In the combined treatment of HIV/AIDS, it can inhibit virus replication by competing with deoxyATP and combining with polymerase in the virus, reduce the virus amount, reduce the attack on CD4 + T lymphocytes, rebuild the immune system, increase the immune lymphocytes, improve the immune kinetic energy, and increase the CD4 + T cell count.^[22,23]^

HIV/AIDS patients can achieve virological suppression and immune reconstitution through effective drug treatment, but basically all drugs can produce adverse reactions in the early stage of treatment, which is one of the reasons for the discontinuation or change of treatment regimen.^[24,25]^ During the 3-month follow-up period of this study, most of the adverse reactions were mild, and the adverse reactions were also at the early stage of the medication, so the patients could recover spontaneously, or tolerate them. Moreover, the adverse effects in the nevirapine group were significantly lower than those in the efavirenz group, suggesting that nevirapine has a safer clinical effect.

Our study has several limitations that should be acknowledged. First, this is a single-center retrospective study with a relatively small sample size (n = 100), which limits the generalizability of our findings to broader HIV/AIDS populations. Second, the follow-up period of 3 months is relatively short and may not capture long-term efficacy and safety outcomes of these treatment regimens. Third, we did not perform comprehensive subgroup analyses based on important clinical factors such as baseline viral load, CD4 + nadir, and route of HIV acquisition, which could potentially influence treatment responses. Fourth, we did not assess medication adherence systematically, which is a critical factor in antiretroviral therapy outcomes. To address these limitations, we are planning a multicenter prospective study with a larger sample size, longer follow-up period, and more comprehensive outcome assessments including viral load measurements and detailed quality of life evaluations.

In conclusion, for the treatment of HIV/AIDS patients at our center, the regimen of lamidudine + zidovudine combined with nevirapine demonstrated superior outcomes compared to the treatment of lamivudine + zidovudine and efavirenz, with significant improvements in CD4 + T lymphocyte counts, enhanced immune function, and fewer adverse reactions. These findings suggest that the nevirapine-containing regimen may be preferable for improving immunological recovery and treatment tolerability in this patient population.

## Acknowledgments

Thanks to all those who helped with the study but were not listed as coauthors due to insufficient contributions.

## Author contributions

**Project administration:** Meiyin Zou.

**Supervision:** Meiyin Zou.

**Writing – original draft:** Xiaohong Zhu.

**Writing – review & editing:** Meiyin Zou.
